# Prevalence of Prostate Cancer Clinical States and Mortality in the United States: Estimates Using a Dynamic Progression Model

**DOI:** 10.1371/journal.pone.0139440

**Published:** 2015-10-13

**Authors:** Howard I. Scher, Kirk Solo, Jason Valant, Mary B. Todd, Maneesha Mehra

**Affiliations:** 1 Genitourinary Oncology Service, Department of Medicine, Sidney Kimmel Center for Prostate and Urologic Cancers, Memorial Sloan Kettering Cancer Center, and Department of Medicine, Weill Cornell Medical College, New York, New York, United States of America; 2 Lexidyne, Colorado Springs, Colorado, United States of America; 3 Janssen Global Services, South Raritan, New Jersey, United States of America; Texas Tech University Health Sciences Center, UNITED STATES

## Abstract

**Objective:**

To identify patient populations most in need of treatment across the prostate cancer disease continuum, we developed a novel dynamic transition model based on risk of disease progression and mortality.

**Design and Outcome Measurements:**

We modeled the flow of patient populations through eight prostate cancer clinical states (PCCS) that are characterized by the status of the primary tumor, presence of metastases, prior and current treatment, and testosterone levels. Simulations used published US incidence rates for each year from 1990. Progression and mortality rates were derived from published clinical trials, meta-analyses, and observational studies. Model outputs included the incidence, prevalence, and mortality for each PCCS. The impact of novel treatments was modeled in three distinct scenarios: metastatic castration-resistant prostate cancer (mCRPC), non-metastatic CRPC (nmCRPC), or both.

**Results and Limitations:**

The model estimated the prevalence of prostate cancer as 2,219,280 in the US in 2009 and 3,072,480 in 2020, and incidence of mCRPC as 36,100 and 42,970, respectively. All-cause mortality in prostate cancer was estimated at 168,290 in 2009 and 219,360 in 2020, with 20.5% and 19.5% of these deaths, respectively, occurring in men with mCRPC. The majority (86%) of incidence flow into mCRPC states was from the nmCRPC clinical state. In the scenario with novel interventions for nmCRPC states, the progression to mCRPC is reduced, thus decreasing mCRPC incidence by 12% in 2020, with a sustained decline in mCRPC mortality. A limitation of the model is that it does not estimate prostate cancer—specific mortality.

**Conclusion:**

The model informs clinical trial design for prostate cancer by quantifying outcomes in PCCS, and demonstrates the impact of an effective therapy applied in an earlier clinical state of nmCRPC on the incidence of mCRPC morbidity and subsequent mortality.

## Introduction

Prostate cancer is a significant cause of morbidity and mortality in the United States. With an estimated incidence of 233,000 new cases and 29,480 deaths in 2014, it is the most frequently diagnosed cancer and second most frequent cause of cancer deaths in US males [1]. Prostate-specific antigen (PSA)-based detection strategies are now widely used in the United States, with the result that most men are diagnosed with the disease clinically confined to the gland [2]. This has also led to earlier intervention and, in parallel, declining mortality, although the overall impact of early detection is controversial [3]. Additionally, for many men diagnosed with prostate cancer, the risk of cancer-related symptoms, metastases, and death from disease is low. A challenge in setting expectations for clinical outcome and reliably assessing prognosis is that prostate cancer is a dynamic disease that changes over time as a function of the intrinsic properties of the tumor, patient factors, and the specific therapies to which the tumor has been exposed.

Understanding the prognosis for patient populations at different points in the prostate cancer disease continuum is essential to guide management and improve patient outcomes, an issue not addressed by traditional staging systems or nomograms. In 2000, we proposed a dynamic progression model that partitioned both the untreated natural history and post-treatment history of the prostate cancer disease continuum from diagnosis to death into distinct clinical states [4]. Each state represents a clinically significant milestone and key decision point that is easily recognized by patients, physicians, and researchers. The dynamic progression of patients through these clinical states across the disease continuum has been described [4–6]. Here we present a dynamic model that quantifies the number of patients diagnosed with prostate cancer in each clinical state using published data on cumulative disease incidence, progression, and mortality. The objective was to develop a disease progression model to identify patient populations most at risk of disease progression and/or mortality and to thus focus clinical research. Used to explore different clinical scenarios, the model can help clarify the impact of novel therapeutics/diagnostics on future prostate cancer disease outlook. In three distinct hypothetical scenarios, we illustrate the impact of introducing novel life-prolonging therapy for non-metastatic and metastatic disease states. We demonstrate that developing interventions for non-metastatic castration-resistant prostate cancer (nmCRPC) should be a priority for clinical research.

## Methods

### Prostate Cancer Clinical States and Data Sources

The eight prostate cancer clinical disease states were updated from the original published description [4] and aligned with treatment algorithms of current clinical practice guidelines based on therapeutic advances [2]. The clinical state definitions are based on the status of the primary tumor, presence or absence of detectable metastases, prior and current treatment (including cytotoxic therapy), and serum testosterone levels (non-castrate/castrate). Individual trials were assigned to a specific state based on the study eligibility criteria. For systemic therapies, only those that had received regulatory approval by 2009 (the final year of model inputs) were included. A detailed definition of each prostate cancer clinical state is provided in Table 1.

**Table 1:** Definitions of prostate cancer clinical states comprising the dynamic transition model.

Clinical state	Definition
Localized Prostate Cancer	
Newly diagnosed; localized disease	• Newly diagnosed prostate cancer localized to the gland
	• Treatment options include radical surgery, radiation therapy (by external beam, internal implants, or a combination of the two), or active surveillance
	• These patients remain in this state after initial treatment until disease recurrence or death. An active surveillance patient who later requires treatment for worsening local disease remains classified in this state until disease recurrence and transition to the locally advanced disease state
	• This state comprises the overwhelming majority of newly diagnosed prostate cancer patients in the United States
Newly diagnosed; locally advanced disease	• Newly diagnosed patients presenting with “high risk” locally advanced tumors that extend beyond the capsule of the prostate (infiltrating neighboring structures and involving regional lymph nodes, without distant metastases) or who have a predicted high probability of recurrence after local therapy alone, based on the combination of T stage, nodal status, Gleason score, and PSA levels at the time of diagnosis [2,7,8]
	• Similar to the newly diagnosed, localized disease state, treatments are directed at the primary tumor (but more typically involve concurrent use of hormone therapies, particularly in patients treated with radiation therapy), and patients have not yet experienced a recurrence of their disease or died. Active surveillance is not appropriate
	• In the United States, this patient population accounts for <20% of the annual incidence of prostate cancer
Rising PSA (non-castrate) (biochemical failure after local therapy)	• Patients who have experienced biochemical failure after receiving local treatment for prostate cancer (i.e., surgery or radiation therapy) with non-castrate levels of testosterone and no detectable disease on imaging tests (bone scan, CT scan, or MRI)
nmCRPC	
Rising PSA (non-castrate) (biochemical failure after hormonal therapy)	• Patients with biochemical failure after local therapy or newly diagnosed, locally advanced disease who have received hormonal therapy and experienced biochemical failure despite castrate levels of testosterone (testosterone <50 ng/dl) with no detectable disease on imaging scans (bone scan, CT scan, or MRI)
Hormone-sensitive, metastatic prostate cancer	
Newly diagnosed; metastatic disease	• Patients who have metastatic disease detectable on imaging (i.e., bone scan, CT scan, or MRI) at the time of first diagnosis. These patients have either not received or are continuing to respond (i.e., are not showing progression) to primary hormone therapy
	• This state currently accounts for <5% of the annual incidence of prostate cancer in the United States [7]
mCRPC	
Asymptomatic/minimally symptomatic mCRPC that has not been treated with or not progressed on chemotherapy	• Patients with mCRPC who have minimal or no symptoms (such as self-reported pain) and have either not received or not progressed on chemotherapy; currently approved therapies include androgen biosynthesis and androgen receptor signaling inhibitors, immunotherapy, and/or docetaxel-based chemotherapy*
Symptomatic mCRPC that has not been treated with or not progressed on chemotherapy	• Patients with mCRPC who have moderate to severe symptoms, have progressed on an androgen biosynthesis inhibitor and/or androgen receptor signaling inhibitor, and have either not received or not failed a chemotherapy treatment
mCRPC that progressed on/after first-line chemotherapy	• Patients with metastatic disease who have failed at least one chemotherapy regimen

*Based on publications of trials focused on patients with or without symptoms.

PSA, prostate-specific antigen; CT, computed tomography; MRI, magnetic resonance imaging; nmCRPC, non-metastatic castration-resistant prostate cancer; mCRPC, metastatic castration-resistant prostate cancer.

Results of selected phase III trials, meta-analyses, and observational studies designed to establish new standards of care for the respective state were also considered (Table 2). The data sources listed in Table 2 focused on large, contemporary, pivotal studies that met level I evidence criteria and impacted current clinical practice in the United States; several of the data sources were cited in the 2015 National Comprehensive Cancer Network guidelines [2]. For early-stage disease, a recent large meta-analysis of pivotal trials in early-stage disease was used [9]. Since Kaplan-Meier curves of overall survival (OS) and progression-free survival (PFS) were required as data inputs for the model, publications that did not contain this information were not considered. Each identified publication was subsequently reviewed in depth and the final selection was determined by how closely the respective patient population matched the defined clinical state. A summary of each publication is provided (Table A in S1 File).

**Table 2:** Data sources used to determine the hazard rates for progression-free survival and overall survival associated with each clinical state, and the survival estimates derived from these publications for inclusion into the model.

Clinical state	Treatment approach	Data source	Overall survival	Progression-free survival
Newly diagnosed prostate cancer; localized disease	No treatment/watchful waiting	Bill-Axelson et al 2008 [10]	5-yr: 0.910	5-yr: 0.730
			10-yr: 0.690	10-yr: 0.585
	Surgery only	Moreira et al 2009 [11]	5-yr: 0.920	5-yr: 0.780
			10-yr: 0.800	10-yr: 0.720
	Radiation only	D’Amico 2006 et al [12]	5-yr: 0.890	5-yr: 0.260
			10-yr: 0.600	10-yr: 0.070
	Hormonal therapy only	Antonarakis et al 2007 [9]	5-yr: 0.704	5-yr: 0.565
			10-yr: 0.487	10-yr: 0.318
Newly diagnosed prostate cancer; locally advanced disease	No treatment/watchful waiting	Shappley et al 2009 [13]	5-yr: 0.636	5-yr: 0.510
			10-yr: 0.354	10-yr: 0.300
	Surgery plus hormonal therapy	Antonarakis et al 2007 [9]	5-yr: 0.888	5-yr: 0.649
			10-yr: 0.791	10-yr: 0.415
	Surgery plus radiation	Bolla et al 2005 [14]	5-yr: 0.908	5-yr: 0.745
			10-yr: 0.821	10-yr: 0.565
	Radiation only	Bolla et al 2002 [15]	5-yr: 0.799	5-yr: 0.452
			10-yr: 0.559	10-yr: 0.093
	Radiation plus hormonal therapy	Antonarakis et al 2007 [9]	5-yr: 0.824	5-yr: 0.525
			10-yr: 0.684	10-yr: 0.276
	Hormonal therapy only	Antonarakis et al 2007 [9]	5-yr: 0.704	5-yr: 0.565
			10-yr: 0.487	10-yr: 0.318
Biochemical failure after local therapy/rising PSA	NA (natural history)	Antonarakis et al 2011 [16]	5-yr: 0.882	5-yr: 0.315
			10-yr: 0.778	10-yr: 0.099
nmCRPC	NA (natural history)*	Smith et al 2011 [17]*	5-yr: 0.350	5-yr: 0.220
			10-yr: 0.002	10-yr: 0.081
Newly diagnosed prostate cancer; metastatic disease	Any (i.e., either no treatment, surgery plus hormonal therapy, radiation plus hormonal therapy, hormonal therapy, or chemotherapy)	Tangen et al 2012 [18]	5-yr: 0.430	-
			10-yr: 0.180	-
	Any (i.e., either no treatment, surgery plus hormonal therapy, radiation plus hormonal therapy, hormonal therapy, or chemotherapy)	Noguchi et al 2004 [19]	-	5-yr: 0.238
			-	10-yr: 0.076
Asymptomatic/minimally symptomatic mCRPC that has not been treated with or has not progressed on chemotherapy	Immunotherapy	Small et al 2006 [20]	5-yr: 0.106	5-yr: 0.000
			10-yr: 0.023	10-yr: 0.000
Symptomatic mCRPC that has not been treated with or has not progressed on chemotherapy	Chemotherapy	Petrylak et al 2004 [21]	5-yr: 0.054	5-yr: 0.000
			10-yr: 0.003	10-yr: 0.000
mCRPC that progressed on/after first-line chemotherapy	NA (natural history)	de Bono et al 2011 [22]	5-yr: 0.016	5-yr: 0.000
			10-yr: 0.000	10-yr: 0.000

*The distribution of patients flowing from nmCRPC to mCRPC that has not been treated with or not progressed on chemotherapy was determined based on Oudard et al 2009 [23].

PSA, prostate-specific antigen; nmCRPC, non-metastatic castration-resistant prostate cancer; mCRPC, metastatic castration-resistant prostate cancer; NA, not applicable.

### Model Summary

The prostate cancer clinical states progression model simulated patient flow through the eight distinct prostate cancer clinical states over time using a novel dynamic modeling framework. These models were custom programmed in the Java programming language (Java SE 6; Oracle Corporation, Redwood City, CA, USA). Additional details on the modeling methodology and the underlying mathematical approach is presented in S1 File.

The base-case model simulated the annual progression of prostate cancer patients through the eight clinical states from diagnosis to death over a 19-year period from 1990 to 2009 (Table 3). The simulation started in 1990 with the diagnosed incidence of prostate cancer patients by stage (localized, locally advanced, and non-castrate metastatic disease); the annual incidence for each subsequent year by stage was then added. In addition, a forward-looking model was developed that used the Surveillance, Epidemiology and End Results Program (SEER) age-specific prostate cancer incidence rate data from 2008 to estimate prostate cancer incidence for each year from 2009 to 2020 (Table 3). To validate the model, the final results were compared with published estimates of prostate cancer incidence and prevalence in the United States for 2009 and 2020 [7,24].

**Table 3:** Annual progression and mortality rates for the base-case model in 2009 and 2020 projections.

	Base-case model 2009			2020 Projections		
Clinical state	Incidence*	Prevalence	Mortality	Incidence*	Prevalence	Mortality
Newly diagnosed prostate cancer; localized disease*	194,765*	1,383,920	73,485	259,715*	2,075,945	10,915
Newly diagnosed prostate cancer; locally advanced disease*	27,555*	199,410	12,845	37,310*	237,515	13,920
Biochemical failure after local therapy/rising PSA**	83,260	446,540	26,510	98,800	528,770	29,725
Biochemical failure after hormonal therapy**	49,390	91,780	15,130	58,960	112,065	18,615
Newly diagnosed prostate cancer; metastatic disease*	9790*	35,520	5795	13,575*	41,495	6565
Asymptomatic/minimally symptomatic mCRPC that has not been treated with or not progressed on chemotherapy**	17,185	6745	2205	20,255	8320	2785
Symptomatic mCRPC that has not been treated with or not progressed on chemotherapy**	30,010	32,145	15,415	36,640	39,650	18,600
mCRPC that progressed on/after first-line chemotherapy**	17,580	23,220	16,905	21,700	28,720	21,235

*Incidence occurs with diagnosis of prostate cancer.

**Incidence is derived from progression from earlier clinical states as shown in Fig 2.

PSA, prostate-specific antigen; mCRPC, metastatic castration-resistant prostate cancer.

The utility of the model as a scenario tool was tested in three hypothetical independent scenarios that evaluated the potential impact of a new treatment in the early nmCRPC and/or mCRPC states. In the early nmCRPC scenario, the novel therapy for nmCRPC has the same OS relative to other nmCRPC therapies, but a 25% improvement in PFS. In the mCRPC scenario, the novel therapy for mCRPC has the same PFS as other mCRPC therapies, but a 25% improvement in OS. In the third scenario (combined), novel therapies were introduced in both nmCRPC and mCRPC states, with the aforementioned assumptions.

## Results

**Fig 1:**
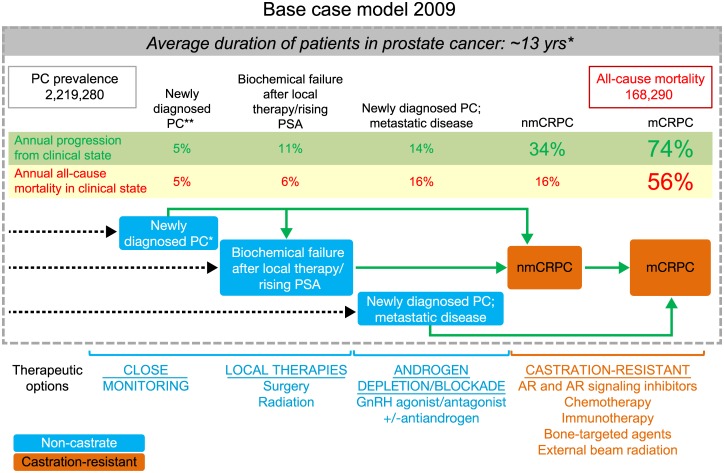
Structure and results of the dynamic progression model depicting patient flow between the distinct prostate cancer clinical states in the base-case model from 1990 to 2009. The model highlights movement to clinical states that have higher mortality rates. Improvement in progression-free survival of men in the nmCRPC scenario reduces the number of patients transitioning from nmCRPC into the mCRPC population, where mortality risk is highest and, as such, has a more permanent impact on mCRPC mortality. *Weighted averages of patents diagnosed with localized, locally advanced, and metastatic disease. **Localized disease or locally advanced disease. nmCRPC, non-metastatic castration-resistant prostate cancer; mCRPC, metastatic castration-resistant prostate cancer.

In 2009, the nmCRPC and mCRPC clinical states associated with castration-resistant disease had higher rates of mortality and annual progression relative to the localized or non-castrate states (Figure A in S1 File). The incidence, prevalence, and mortality associated with the distinctive clinical states in the current (base year 2009) and future (2020) prostate cancer landscape are shown (Table 3, Fig 1). Based on the 19-year simulation, the base-case model estimated a prevalence of 2,219,280 men in the United States diagnosed with prostate cancer in 2009 (Fig 1). Of these, 2,121,650 (95.6%) presented with localized or locally advanced disease while 97,630 (4.4%) had metastatic prostate cancer (corresponding to non-castrate and mCRPC states). The base model estimated an mCRPC incidence of 36,100 in 2009. The nmCRPC clinical state contributed heavily to the mCRPC states (86%), while the incidence flow from the non-castrate state to mCRPC was <15%. All-cause mortality in 2009 in the base-case model was estimated at 168,290 deaths, with 34,525 or 20.5% of these deaths in patients in mCRPC states.

The model estimates for the year 2020 are based on existing/current (2009) disease incidence, diagnosis, and treatment patterns, and reflect demographic changes in the US population over time (e.g., the impact of the baby boomer population) (Table 3). Corresponding estimates for each year between 2009 and 2020 are shown in Table B in S1 File. Notably, the total prevalence of patients with prostate cancer is estimated to increase to 3,072,480. In 2020, the model estimated mCRPC incidence at 42,970 cases. Similar to the base-case model, the majority of the mCRPC incidence was derived from the nmCRPC state (86%, or 36,870 cases), with <15% from the non-castrate state. All-cause mortality in 2020 in the base-case model was estimated at 219,360 deaths, with 19.5% (or 42,680) of deaths occurring in the mCRPC states.

**Fig 2:**
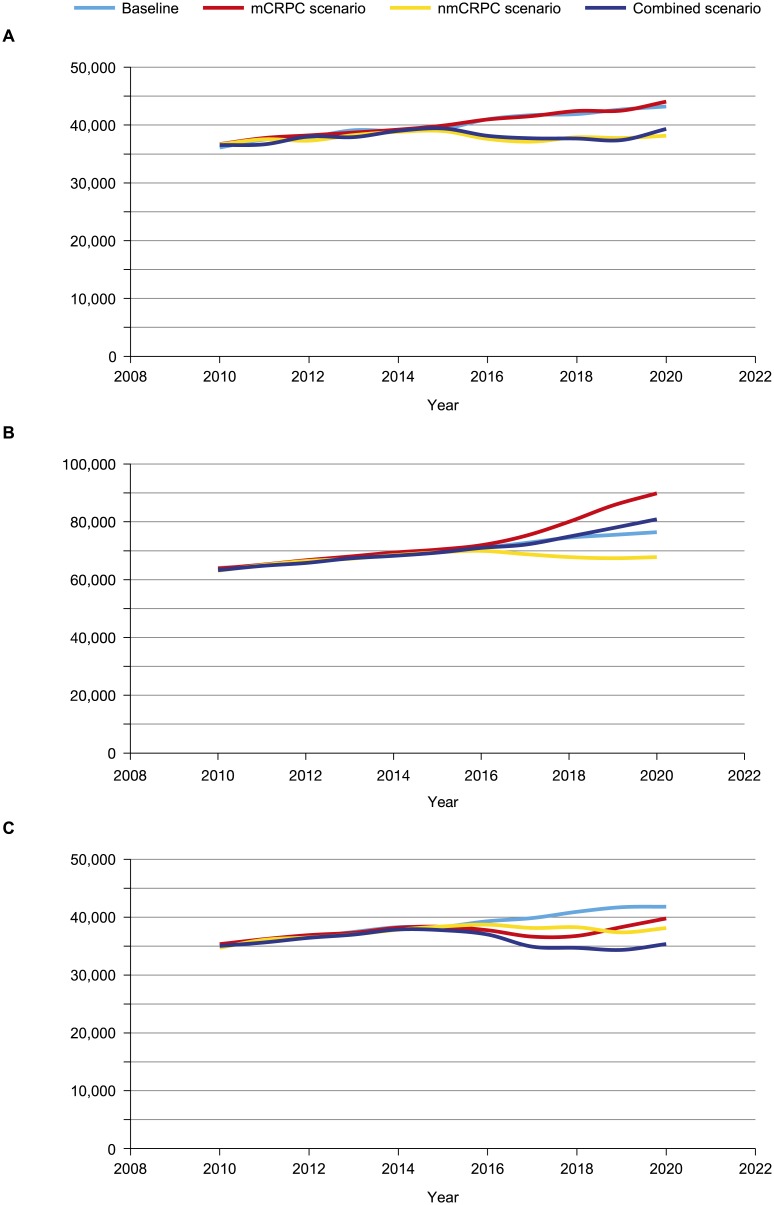
Impact of hypothetical novel treatment in 2015 for early-stage nmCRPC, mCRPC, and combined scenario. (A) mCRPC incidence, (B) mCRPC prevalence, and (C) mCRPC mortality. The reduction in mCRPC incidence would lead to lower mCRPC prevalence and a sustained decline in mCRPC mortality. nmCRPC, non-metastatic castration-resistant prostate cancer; mCRPC, metastatic castration-resistant prostate cancer.

To illustrate the practical applicability of the model, we estimated the impact of novel treatments in three scenarios: improved PFS in early nmCRPC, improved OS in mCRPC, and a combined scenario with improvements in PFS and OS for nmCRPC and mCRPC, respectively. In the nmCRPC scenario, a novel therapeutic agent associated with improved PFS introduced in 2015 results in lowering mCRPC incidence by 11.7% (5061 patients less) in 2020 (Table 4) compared with the 2020 baseline mCRPC incidence of 43,211. This reduction in mCRPC incidence leads to a lower mCRPC prevalence and, hence, a sustained decline in mCRPC mortality (Fig 2). With improved PFS, prevalence of nmCRPC will be greater by 12% (13,922 more patients) relative to the 2020 baseline prevalence (Table 4) of 112,410 patients. In the mCRPC scenario, a novel therapeutic introduced in 2015 associated with improved OS reduces mCRPC mortality, with 2032 fewer deaths in 2020 and a simultaneous increase in mCRPC prevalence of 13,448 patients (Table 4). With the increase in mCRPC prevalence over time (due to the reduction in annual mortality outflow rate from 54% to 44% from the mCRPC clinical state), the volume/number of deaths after an initial decline will subsequently increase (Fig 2C). Finally, the combined scenario (simultaneous introduction of novel therapy in clinical state nmCRPC and mCRPC) results in a reduction of more than 6000 deaths from mCRPC in 2020.

**Table 4:** Outcomes in hypothetical scenarios: novel therapy introduced in 2015 for early nmCRPC, mCRPC, or combined scenarios.

	2017				2020			
	Baseline	mCRPC scenario	nmCRPC scenario	Combined scenario	Baseline	mCRPC scenario	nmCRPC scenario	Combined scenario
mCRPC incidence	41,721	NA	37,131	37,708	43,211	NA	38,150	39,327
mCRPC prevalence	72,677	75,132	68,837	72,115	76,431	89,879	67,796	80,894
mCRPC mortality	39,870	36,657	38,156	34,926	41,833	39,801	38,139	35,370
nmCRPC	107,124	NA	109,540	110,954	112,410	NA	126,332	126,744

mCRPC, metastatic castration-resistant prostate cancer; nmCRPC, non-metastatic castration-resistant prostate cancer; NA, not applicable.

## Discussion

This dynamic clinical states transition model for the United States estimated the point prevalence of prostate cancer as 2.2 million in 2009, which will increase to 3.07 million in 2020. These prevalence totals are the cumulative result of the inflow of newly diagnosed patients and outflow of patients who succumb from 1990 onward. A change in the input variables for the purpose of calibration was avoided in order to maintain a purely data-driven model. To ensure the accuracy of the cumulative flow approach, the results were compared and validated against reported epidemiological prevalence and mortality data. The SEER reported complete prevalence (>35 years’ duration) for prostate cancer of 2.3 million in 2009 (vs. 2.2 million simulated) and the National Institutes of Health projected 3.11 million (vs. 3.07 million simulated) in 2020 in the trend incidence scenario [24]. The prevalence for 2009 was lower than that reported by SEER, in part because it did not account for prostate cancer incident cases diagnosed prior to 1990 [7]. The all-cause mortality of 168,290 deaths in 2009, with 34,525 deaths in the mCRPC population, projected by the model approximates the annual prostate cancer mortality of 30,000 reported by the American Cancer Society [25].

Coupling (or using) the estimate of the number of patients at different points in the disease continuum along with what is known about the frequency of prostate cancer—specific morbidities that occur in a given state enables (i) a more global estimate of the morbidity and adverse impact of the disease, and (ii) the quantification of outcomes (mortality and progression) with the introduction of novel therapies for specific clinical states across the disease continuum. The information provides valuable insights into unmet needs short of an improvement in survival being the focus of future or new drug development efforts in a rapidly changing therapeutic landscape. The mCRPC state in which effective treatments were introduced with a targeted mortality reduction threshold of 25% in registration trials revealed a reduced mortality risk and substantially reduced mCRPC mortality, with ~2000 fewer deaths projected in 2020 (Table 4). The decline in mCRPC mortality was temporary until the model reached equilibrium, which occurred quickly given the survival benefit of only 4 months.

The advantage of applying an effective therapy earlier in the disease was shown by modeling the nmCRPC state. The model demonstrates that reducing the incidence of mCRPC with such an intervention would have a greater and more sustained impact on mCRPC mortality and morbidity. Based on prevalence, progression, and mortality associated with nmCRPC, men in this state of the disease represent a patient population for whom preventing or delaying a transition to mCRPC is a primary therapeutic objective, which could have a significant impact from a public health perspective. Importantly, the ability of the model to demonstrate an improvement in outcomes through a development program focused on a specific prostate cancer clinical state also provides a justification for committing the resources needed to complete such trials. Along these lines, it is notable that several clinical trials in this population are ongoing (Clinical Trials.gov: NCT01946204, NCT02003924, NCT01046916, NCT01703065, NCT01875250) and, once they are completed, their impact on disease mortality could be estimated using this dynamic model.

As expected, the model also shows that all-cause mortality rates in prostate cancer patients increase with disease progression as defined by the clinical state. Patients with mCRPC in particular have a high risk of mortality and progression. While prostate cancer—specific mortality is not specified in this model and this is a limitation, the increase in mortality rate reported in the 2009 base model can be attributed to the increasing proportion of deaths due to or attributed to prostate cancer given the following reasoning: Based on US Census data, the all-cause mortality rate among males aged 65 and older was 5.67% in 2010 [26]. The average age of patients who are diagnosed with prostate cancer is 67 years [7] and, based on our 2009 model, the all-cause mortality rate among patients with prostate cancer is 7.6%. In the early stages of disease, the mortality rate of 5.7% is similar to the all-cause mortality rate in the US population aged 65 and older, indicating that only a small proportion (if any) of deaths in these clinical states is due to prostate cancer while the mortality rate of 55.3% for mCRPC is much higher (Figure B in S1 File). Applying the ratio of US all-cause mortality rate to the mCRPC mortality rate suggests that 90% (or ~31,000) of the 34,525 deaths in mCRPC patients can be attributed to prostate cancer, thus aligning with published estimates of annual prostate cancer—specific mortality in the United States. With regard to new prostate cancer therapies, OS will ultimately encompass both all-cause mortality rates and prostate cancer—specific mortality rates (with a delta between the two survival rates). In the model, the relationship between an improvement in PFS vs. OS is quantified and demonstrates the benefits and limitations of these two measures in the nmCRPC and mCRPC scenarios. Improvement in PFS of men in the nmCRPC scenario reduces the number of patients transitioning from nmCRPC into the mCRPC population, where mortality risk is highest. Thus, the ability to reduce patient transitions to a clinical state with higher mortality is based on the ability of new therapies to extend the duration of survival without disease progression (i.e., PFS). Improvements in PFS and OS in the mCRPC scenario essentially delay the inevitable, as following the initial reduction in mortality the levels rebound close to the base case. Thus, the nmCRPC scenario supports the use of PFS as an end point for trials in patient populations whose all-cause mortality outweighs prostate cancer—specific mortality, yet who have a higher risk of progression. These observations are consistent with the Prostate Cancer Clinical Trials Working Group recommendations [27].

The existing model framework can be adapted to quantify changes in disease mortality and morbidity as a result of novel treatments or interventions, such as screening and diagnostics or for specific countries and different timeframes. In Europe, for example, a much higher proportion of patients present with metastatic disease at the time of diagnosis and the time of progression from the non-castrate state to mCRPC is generally much shorter [28]. Since this transition model quantifies the progression from early to more advanced disease states, it could help assess the overall impact of changes in routine PSA screening in Europe and the United States.

## Conclusion

We describe the first dynamic clinical states transition model that provides a quantitative assessment of the US prostate cancer disease landscape defined by eight distinct clinical states. The model indicates that US patients with nmCRPC and mCRPC are the populations in greatest need for new, more effective treatment options that prolong survival or delay disease progression. We also demonstrate the model’s ability to predict the future epidemiology of prostate cancer with the introduction of novel therapies, and to inform clinical trial design. These results also provide a framework to validate and qualify PFS as an end point that can lead to regulatory approvals and accelerate drug development.

## Supporting Information

S1 File Additional details on modeling methodology. Summary of publications used as data sources for the clinical states model (**Table A**). Prevalence of clinical states, incidence flow, and patient flows between the clinical states for each year from 2010 to 2020 (**Table B**). Incidence of prostate cancer in the United States between 1990 and 2009. Grouped by clinical state at the time of diagnosis according to the Surveillance Epidemiology and End Results database (**Figure A**). Annual all-cause mortality by clinical state, base-case model in 2009 (**Figure B**).
(DOCX)
