# Supplementary material for: Prevalence of Prostate Cancer Clinical States and Mortality in the United States: Estimates Using a Dynamic Progression Model
Source: PLoS One. 2015 Oct 13;10(10):e0139440. doi: 10.1371/journal.pone.0139440 (PMC4603789; doi:10.1371/journal.pone.0139440)
Supplement: S1 File — Summary of publications used as data sources for the clinical states model (Table A). Prevalence of clinical states, incidence flow, and patient flows between the clinical states for each year from 2010 to 2020 (Table B). Incidence of prostate cancer in the United States between 1990 and 2009. Grouped by clinical state at the time of diagnosis according to the Surveillance Epidemiology and End Results database (Figure A). Annual all-cause mortality by clinical state, base-case model in 2009 (Figure B). (DOCX) [file pone.0139440.s001.docx]

# SUPPORTING INFORMATION: Additional Details on Modeling Methodology

**Prevalence of Prostate Cancer Clinical States in the United States:**

**Estimates Using a Dynamic Progression Model**

*Howard I. Scher^1*^, Kirk Solo^2^, Jason Valant^2^, Mary B. Todd^3^, Maneesha Mehra^3^*

^1^ Genitourinary Oncology Service, Department of Medicine, Memorial Sloan Kettering Cancer Center, and Department of Medicine, Weill Cornell Medical College, New York, New York, United States of America

^2^ Lexidyne, Colorado Springs, Colorado, United States of America

^3^ Janssen Global Services, South Raritan, New Jersey, United States of America

* Corresponding author

E-mail: [scherh@mskcc.org](mailto:scherh@mskcc.org)

# Additional Details on Modeling Methodology

## [Model](#_Toc225602748) Overview

Patient populations within each clinical state represented point prevalence (the number of patients in a state at any given year of the simulation). For each clinical state, patient inflows were determined by the annual diagnosed incidence at presentation, and patient outflows by the estimated number of patients who transitioned from one clinical state to another (disease progression) and by mortality. In this model, prevalence excludes men with undiagnosed cancers known to be present in the population at large.

Each clinical state was assigned an annual all-cause mortality rate, and deceased patients were treated as model outflows exiting the simulation. As such, the point prevalence for each state represented the sum of patients already in that state (those who did not progress or die or were in remission) and newly diagnosed (“incident”) patients.

The annual rates for progression and mortality in the model varied over time as a function of the clinical state and of the type and duration of treatment the patient received. The annual progression between clinical states and the mortality rates for each clinical state were derived from published Kaplan-Meier curves of progression-free survival and overall survival, respectively, as reported from clinical trials (in the form of primary analyses based on prespecified protocols, post hoc analyses, and/or meta-analyses) or observational studies (Table S2). The hazard rates for each particular combination of clinical state and type of treatment were informed by only one recently published data source; for instance, the hazard rates for patients with newly diagnosed, localized disease treated with surgery plus hormonal therapy were derived from a trial reported in 2006 [1].

Annual incidence of patients with prostate cancer (PC) was entered into the model using age-specific PC incidence rates reported by the Surveillance Epidemiology and End Results (SEER) database [2] and applied to age-based population distributions for each year sourced from US Census Bureau historical and projected data (Fig. S1). The 1992 age-specific rates from the SEER data were used to estimate incidence of PC in 1990 and 1991. Similarly, 2008 age-specific rates from the SEER data were used to estimate incidence in 2009. These untreated, newly diagnosed patients were categorized into clinical state L1 (newly diagnosed, localized disease), L2 (newly diagnosed, locally advanced disease), or M1 (newly diagnosed, metastatic disease); the distribution of the total incident patients among these clinical states for each year was also based on data obtained from SEER (Fig. S1). Within each of these clinical states, the distribution of specific treatment allocation following diagnosis was derived from the National Cancer Database [3] and from Janssen data on file. Patients in L1, L2, or M1 who had disease or treatment progression were assigned to all other clinical states (as defined).

For the models, the inflows into clinical states R1 (biochemical failure after local therapy/rising PSA), M0 (nmCRPC), M2 (asymptomatic/minimally symptomatic mCRPC that has not been treated with or not progressed on chemotherapy), M3 (symptomatic mCRPC that has not been treated with or not progressed on chemotherapy), and M4 (mCRPC that progressed on/after first-line chemotherapy) were the result of disease progression only, with a variable annual rate assigned to each clinical state except state M4. Over the course of each year, a proportion of patients progressed to a more advanced state. For example, patients could progress from localized disease (L1) to a biochemical recurrence (R1) or to metastatic castration resistant disease (M2 or M3) following a second biochemical recurrence (M0). Patients did not move backward even if the disease had been eliminated; they only moved forward between states. Of note, patient cycling could occur within the individual clinical states M3 and M4 (but not between these two states) and was determined by the state definitions (see Table 1 in the main article for details).

## Model Development

To support a PC disease progression model that includes the natural and treated history of PC, it was necessary to consider the disease at a virtual patient level in a clinical states context. Each state represents a key decision point for which specific therapeutic approaches, including no therapy, are advised. To achieve this, a software simulation approach termed agent-based modeling was used. This well-established modeling approach simulates virtual entities and allows for conditional probabilities of future behavior to be based on an individual’s history and attribute segmentation.

A bootstrap methodology was implemented to develop a prevalent population made up of all surviving patients who had been diagnosed from 1990 through 2009. Newly diagnosed patients entered initial lines of therapy and progressed based on progression and survival data specific to their clinical state and treatment modality. These virtual patients aged as the simulation progressed. This approach allowed for a 19-year simulation initiated in 1990. The flexibility of the simulation approach also allows for forecast simulations into the future, based on epidemiologic and treatment knowledge and assumptions.

The modeling approach used a dynamic simulation of PC epidemiology that implemented the relationship to diagnosed incidence rates. By applying age-based diagnosed incidence rates to US Census Bureau age distributions over time, it became possible to determine single age cohort incidence volumes over time. The simulation approach allowed for the creation of virtual patients who are allocated to specific age cohorts, who aged each year of the simulation until death.

Once a virtual patient had been created and added to the incident population, currently available data relating to historical treatment segmentation were applied to the incident patient flow and patients were allocated on a probabilistic basis to one of several initial clinical states. After a patient had been allocated to a clinical state and the associated treatment, the individual was assigned a specific progression and mortality risk based on published Kaplan-Meier evaluations from key studies. The time since treatment initiation was tracked and hazard rates were adjusted accordingly, thus allowing for differences in hazard rates based on treatment duration.

Disease progression and mortality were determined by applying random draws against the hazard rates described. If an individual failed a progression hazard draw, he was determined to have failed his current treatment and was advanced to the next clinical state and corresponding therapy; this therapy was determined based on the treatment history and a probabilistic allocation across all next available therapies. When a patient failed a mortality hazard draw, he was removed from the model and included in that year’s all-cause mortality for the clinical state in question. Furthermore, there was an age-based mortality factor that was integrated into the overall survival dynamic.

Patient volumes could be calculated and allocated across a number of different data elements, including clinical state, treatment, and age cohort, allowing for a very flexible representation of the overall population dynamic.

## [PC Inputs and Equations](#_Toc225602748)

The model’s dynamics are based on a system dynamics conceptualization that has been implemented in a discrete fashion using agent-based simulation methodologies. At a simple level, the model is a series of partial differential equations, as shown below in *Equation 1* and *Equation 2.* This can be illustrated by a simple stock/flow representation of the aggregate epidemiology of the disease as shown below. Clinical state outflow can also include progression.

(Stock)

= Prevalence

Inflow

Outflow

$I$= Incidence $M$= Mortality

The key inputs associated with the macro-level epidemiologic dynamics can be simplified into a few generalized data dynamics. Overall incidence is based on age-based incidence rates applied to the overall age distribution of males in the United States. This same approach can be extrapolated to epidemiologic data from other countries. Overall mortality is based on clinical state and treatment-specific survival curves.

Movement between clinical states is determined by a combination of disease progression survival/hazard and overall survival/hazard by therapy and clinical state.

Key epidemiologic inputs include:

- Age-based incidence
- Population by age by year

Key treatment dynamics include:

- Disease progression survival/hazard by therapy/clinical state
- Overall (mortality) survival/hazard by therapy/clinical state
- Distribution of therapies for a given year and clinical state

## Patient Flow

The differential equation representation of the total stock of PC patients (*Equation 1*) in the United States is a function of a constant multiplied by the integral of the difference in inflow and outflow rates. The change in the rate of the stock of patients (*Equation 2*) is a function of the difference of the inflow and outflow rates divided by the determined constant.

Equation 1

$$P=\frac{1}{A}\int\left( I-M \right)dt$$

Equation 2

$$\frac{dP}{dt}=\frac{I-M}{A}$$

## Patient Summation

A summation representation of the above formulae can be represented as follows, where P is prevalence, I is incidence, M is mortality, Rate is age-based incidence rate, and Pop is age cohort population:

Equation A:

$$P=\sum_{age}^{(yr)} \left( P_{age(yr-1)}+I_{age(yr-1)}-M_{age(yr-1)} \right)$$

Equation B:

$$I=\sum_{age \& year} \left( ({Rate}_{age\&year})({Pop}_{age\&year}) \right)$$

The following graphical representation illustrates the overall dynamics:


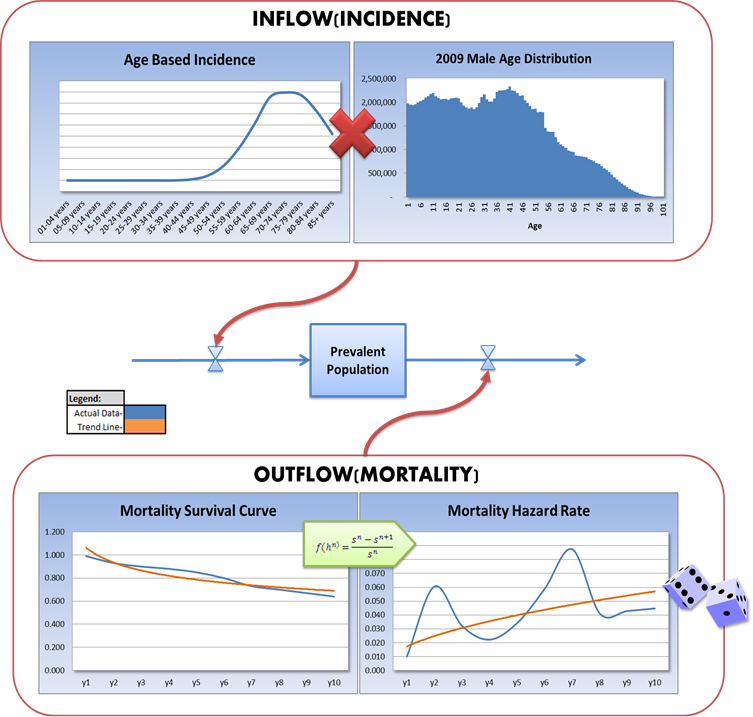


(INFLOW) Incidence =

$$F\left( P,I \right)=P\left( a \right)*I$$

$P=$ Population, a function of age ($a$)

$I=$ Incidence rate

(OUTFLOW) Hazard Function =

$$h^{n}=f\left( \frac{s^{n}-s^{n+1}}{s^{n}} \right)$$

$h^{n}=$ Hazard rate at time n

$s^{n}=$ Survival rate at time n

$s^{n+1}=$Survival rate at time n+1

The dynamics of an individual clinical state can be defined in a similar fashion:


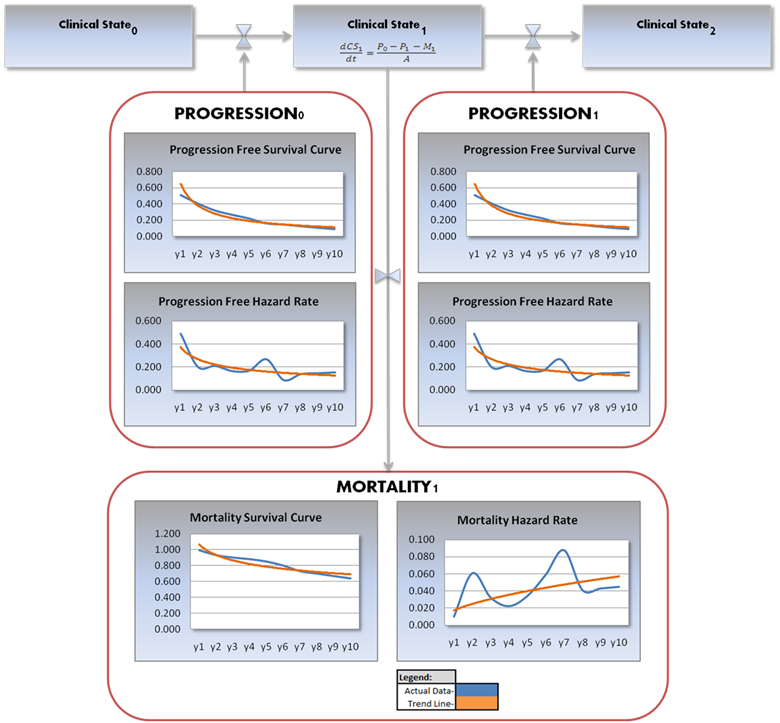


Each individual clinical state may have multiple inflows and outflows that influence the model, all of which can be represented as a partial differential equation (shown above) that is driven by the composite dynamics of the underlying virtual patients.

## [Population and Epidemiology](#_Toc225602749)

Population data are based on current single age cohort male populations and future population estimations for the years 1990 through 2020, as provided by the 2000 Census from the US Census Bureau. This approach allows for the incorporation of age-based demographic shifts on yearly incidence. Historical age-based incidence rates were sourced from the SEER database starting in 1990 and through to 2008. Forward-looking age-based incidence rates were assumed to remain constant, based on the most recently available data from SEER.

By combining the age-based population distributions with the age-based incidence rates, it becomes possible to establish yearly incidence volume by individual age cohort. This establishes the incident diagnosed portion of the disease model, which can then be directly compared with published estimates of PC in the United States.

The mortality aspect of the model’s epidemiology dynamic is based on the overall survival hazard associated with each treatment modality within each of the clinical states. This provides an overall mortality by clinical state, allowing for a comparison of metastatic stage mortality with other published mortality estimates.

The overall prevalence is a function of the bootstrapped incidence and mortality dynamics. The dynamic is a pseudo partial differential equation that results in a 19-year prevalence starting in 2009 and adding a year each year going forward until 2020, at which time a 30-year prevalence is represented by the model.

**PC Diagnosis and Treatment**

The model establishes PC incidence as diagnosed incidence, and as such, the epidemiologic assumptions drive the diagnosis of novel patients. At the point of initial diagnosis, the patient population is apportioned to one of three groups, based on published SEER data:

1. Those with localized disease
2. Those with locally advanced disease
3. Those with metastatic disease

Once allocated to one of these three groups, patients will be probabilistically assigned to either a specific treatment modality or to watchful waiting. “Localized disease” patients will enter clinical state L1, “locally advanced disease” patients will enter L2, and those patients diagnosed with “metastatic disease” will enter M1.

## [Post-treatment Failure and Progression Rates](#_Toc225602752)

Each treatment modality within each segment has an associated yearly progression hazard as well as a yearly mortality hazard, both of which are tied to the amount of time that these patients have been receiving the particular treatment within that specific segment. The progression hazard and mortality hazard for each segment were derived from overall survival and progression-free survival Kaplan-Meier curves from relevant published trials and observational studies (Table S1, as well as Table 1 in the main article). The model applies a probabilistic draw against the hazard rate adjusted to a monthly basis to determine when a patient either advances or dies.

This methodology is a Kaplan-Meier approach applied to an agent-based modeling that allows for differentiated progression and mortality rates based on therapy, time on therapy, and duration that a patient remained in a specific clinical state. There is also a supplemental dynamic tied to baseline age-based mortality that is incorporated into the overall survival dynamic.

## [Model Programming](#_Toc225602753)

The model was developed using custom-coded Java-based functions implemented within a specific simulation environment, both proprietary to Lexidyne (Colorado Springs, CO, USA). Object representations of virtual patients followed a Markov chain–like progression based on survival curves identified for each possible combination of clinical state and assigned treatment. In addition to the clinical state structure, the model also incorporated other patient attributes, such as current treatment, time on therapy, treatment history, and age.

## [Model Estimation and Comparison](#_Toc225602754)

The model explicitly incorporated the core dynamics of PC epidemiology at both aggregate and segmented, or individual, levels. Due to the fact that the model was based primarily on published data, the calibration process was largely limited to a comparison of the results with published estimates for patient population subsets, as available.

### **Table A.** Summary of publications used as data sources for the clinical states model.

| **Publication** | **Sample size** | **Patient recruitment dates** | **Study design** | **Primary end point** | **Median follow-up Time** |
| --- | --- | --- | --- | --- | --- |
| Bill-Axelson et al. 2008 [4] | 348 | 1989–1999 | Randomized clinical trial | Death due to PC | 10.8 years |
| Moreira et al. 2009 [5] | 1156 | 1998–2008 | Retrospective cohort study | PSA persistence | 48 months |
| D’Amico et al. 2006 [6] | 358 | 1989–2002 | Randomized clinical trial | Death due to PC | 4.0 years |
| Shappley et al. 2009 [7] | 174 | 1986–2007 | Prospective observational study | Time to metastasis, time to death | 7.7 years (mean) |
| Antonarakis et al. 2007 [8]  (Hormone therapy as adjunct to radiation therapy) | 5987 | 1987–2003 | Meta-analysis of 16 clinical trials (10 randomized studies, six observational studies) | Overall survival and disease-free survival | 2.6–7.8 years |
| Antonarakis et al. 2007 [8]  (Hormone therapy as adjunct to prostatectomy) | 1885 | 1986–2002 | Meta-analysis of 11 clinical trials (eight randomized studies, three observational studies) | Overall survival and disease-free survival | 2.8–10.4 years |
| Antonarakis et al. 2007 [8]  (Hormone therapy as standalone therapy) | 3233 | 1986–2003 | Meta-analysis of 10 clinical trials (seven randomized studies, three observational studies) | Overall survival and disease-free survival | 3.9–10.4 years |
| Bolla et al. 2005 [9] | 502 | 1992–2001 | Randomized, controlled clinical trial | Biochemical progression-free survival | 5 years |
| Bolla et al. 2002 [10] | 208 | 1987–1995 | Randomized, controlled clinical trial | Disease-free survival | 66 months |
| Antonarakis et al. 2011 [11] | 450 | 1981–2010 | Retrospective analysis | Metastasis-free survival | 8.0 years after prostatectomy, 4.0 years after PSA recurrence |
| Smith et al. 2011 [12] | 331 | 2001–2003 | Post hoc analysis of randomized, controlled clinical trial (placebo group only) | Time to bone metastasis, overall survival, bone metastasis-free survival | N/A |
| Tangen et al. 2012 [13] | 3096 | 1985–2009 | Post hoc pooled analysis of three sequential randomized, controlled clinical trial | Overall survival | N/A |
| Noguchi et al. 2004 [14] | 57 | 1995–1998 | Randomized, controlled clinical trial | Overall response rate, time to objective progression | 26 months |
| Small et al. 2006 [15] | 127 | 2000–2001 | Randomized, controlled clinical trial | Time to disease progression | N/A |
| Petrylak et al. 2004 [16] | 338 | 1999–2003 | Randomized, controlled clinical trial | Overall survival | 32 months |
| de Bono et al. 2011 [17] | 1,195 | 2008–2009 | Randomized, controlled clinical trial | Overall survival | 12.8 months |

PC, prostate cancer; PSA, prostate-specific antigen.

### **Table B.** Prevalence of clinical states, incidence flow, and patient flows between the clinical states for each year from 2010 to 2020.

| **Clinical state*** | **2009** | **2010** | **2011** | **2012** | **2013** | **2014** | **2015** | **2016** | **2017** | **2018** | **2019** | **2020** |
| --- | --- | --- | --- | --- | --- | --- | --- | --- | --- | --- | --- | --- |
| L1 | 1,383,920 | 1,435,490 | 1,490,265 | 1,549,085 | 1,610,605 | 1,674,080 | 1,736,935 | 1,804,005 | 1,871,290 | 1,939,090 | 2,006,800 | 2,075,945 |
| L2 | 199,410 | 199,625 | 200,240 | 202,785 | 205,045 | 207,520 | 211,550 | 215,360 | 221,195 | 226,435 | 232,225 | 237,515 |
| R1 | 446,540 | 454,845 | 462,215 | 466,925 | 472,505 | 478,125 | 48,3205 | 491,050 | 498,475 | 509,350 | 518,215 | 528,770 |
| M0 | 91,780 | 95,075 | 96,895 | 99,225 | 100,650 | 102,405 | 104,585 | 105,235 | 106,505 | 108,145 | 110,555 | 112,065 |
| M1 | 35,520 | 34,380 | 34,375 | 34,590 | 34,720 | 35,215 | 36,415 | 37,170 | 37,900 | 38,930 | 40,110 | 41,495 |
| M2 | 6,745 | 6,870 | 6,955 | 7,290 | 7,385 | 7,595 | 7,445 | 7,660 | 7,595 | 7,925 | 8,305 | 8,320 |
| M3 | 32,145 | 32,920 | 34,040 | 35,000 | 34,970 | 35,530 | 36,035 | 37,025 | 37,295 | 37,940 | 38,250 | 39,650 |
| M4 | 23,220 | 23,895 | 24,390 | 24,700 | 25,545 | 25,790 | 26,415 | 27,200 | 27,800 | 28,115 | 28,940 | 28,720 |
| Total | 2,219,280 | 2,283,100 | 2,349,375 | 2,419,600 | 2,491,425 | 2,566,260 | 2,642,585 | 2,724,705 | 2,808,055 | 2,895,930 | 2,983,400 | 3,072,480 |
| Clinical state | 2009 | 2010 | 2011 | 2012 | 2013 | 2014 | 2015 | 2016 | 2017 | 2018 | 2019 | 2020 |
| L1 | 194,765 | 199,060 | 205,180 | 211,315 | 217,620 | 222,630 | 229,775 | 235,470 | 241,415 | 247,315 | 254,210 | 259,715 |
| L2 | 27,555 | 29,000 | 29,490 | 30,275 | 30,890 | 32,375 | 32,605 | 34,425 | 34,800 | 35,975 | 35,845 | 37,130 |
| M1 | 9,790 | 10,395 | 10,575 | 10,730 | 11,005 | 11,745 | 11,895 | 11,750 | 12,665 | 12,955 | 13,305 | 13,575 |
| Total | 232,110 | 238,455 | 245,245 | 252,320 | 259,515 | 266,750 | 274,275 | 281,645 | 288,880 | 296,245 | 303,360 | 31, 420 |
| From state | To state | 2009 | 2010 | 2011 | 2012 | 2013 | 2014 | 2015 | 2016 | 2017 | 2018 | 2019 |
| L1 | R1 | 69,710 | 68,785 | 67,750 | 68,810 | 69,580 | 71,010 | 72,445 | 74,135 | 77,825 | 78,745 | 80,560 |
| L2 | R1 | 13,550 | 13,925 | 13,730 | 13,970 | 14,410 | 14,230 | 14,655 | 14,535 | 14,945 | 15,255 | 15,560 |
| L2 | M0 | 945 | 975 | 925 | 1035 | 910 | 1,100 | 1,055 | 1,155 | 1,105 | 1,135 | 1,225 |
| R1 | M0 | 48,445 | 48,625 | 49,875 | 50,035 | 51,055 | 52,265 | 51,650 | 52,565 | 53,555 | 55,805 | 56,265 |
| M0 | M2 | 16,920 | 17,630 | 17,745 | 17,930 | 18,605 | 18,485 | 19,075 | 19,295 | 19,160 | 20,035 | 20,005 |
| M0 | M3 | 14,045 | 14,550 | 14,870 | 15,015 | 14,950 | 15,515 | 15,880 | 15,855 | 16,125 | 16,665 | 17,240 |
| M1 | M2 | 265 | 285 | 245 | 185 | 270 | 270 | 295 | 265 | 300 | 300 | 270 |
| M1 | M3 | 4,870 | 4,835 | 4,685 | 4,710 | 4,700 | 4,785 | 5,230 | 5,210 | 5,295 | 5,460 | 5,360 |
| M2 | M3 | 11,095 | 11,385 | 11,855 | 11,505 | 12,015 | 12,300 | 12,295 | 12,595 | 12,440 | 12,805 | 13,320 |
| M2 | M4 | 3,760 | 4,155 | 3,825 | 4,160 | 4,130 | 4,420 | 4,445 | 4,425 | 4,390 | 4,595 | 4,530 |
| M3 | M4 | 13,820 | 13,860 | 14,385 | 14,810 | 15,225 | 14,955 | 15,500 | 16,380 | 16,130 | 16,590 | 16,490 |
| Clinical State | 2009 | 2010 | 2011 | 2012 | 2013 | 2014 | 2015 | 2016 | 2017 | 2018 | 2019 | 2020 |
| L1 | 73,485 | 75,500 | 78,610 | 80,985 | 84,565 | 88,765 | 90,260 | 94,050 | 95,790 | 100,860 | 104,505 | 107,915 |
| L2 | 12,845 | 13,485 | 12,290 | 13,010 | 13,095 | 13,015 | 13,085 | 12,900 | 13,510 | 13,795 | 13,770 | 13,920 |
| R1 | 26,510 | 26,715 | 26,895 | 27,165 | 27,315 | 27,895 | 27,605 | 28,680 | 28,340 | 29,330 | 29,300 | 29,725 |
| M0 | 15,130 | 15,600 | 15,855 | 16,700 | 16,655 | 17,185 | 17,100 | 17,300 | 17,735 | 17,830 | 18,735 | 18,615 |
| M1 | 5,795 | 5,280 | 5,430 | 5,705 | 5,540 | 5,490 | 5,615 | 5,545 | 6,040 | 6,015 | 6,290 | 6,565 |
| M2 | 2,205 | 2,290 | 1,975 | 2,355 | 2,520 | 2,185 | 2,415 | 2,605 | 2,300 | 2,555 | 2,410 | 2,785 |
| M3 | 15,415 | 15,790 | 16,065 | 16,450 | 15,880 | 17,140 | 16,915 | 17,010 | 17,085 | 18,030 | 18,030 | 18,600 |
| M4 | 16,905 | 17,520 | 17,900 | 18,125 | 19,110 | 18,750 | 19,160 | 20,205 | 20,205 | 20,360 | 21, 240 | 21,235 |
| Total | 168,290 | 172,180 | 175,020 | 180,495 | 184,680 | 190,425 | 192,155 | 19,295 | 201,005 | 208,775 | 214,280 | 219,360 |

*L1, newly diagnosed, localized disease; L2, newly diagnosed, locally advanced disease; R1, biochemical failure after local therapy/rising PSA; M0, nmCRPC; M1, newly diagnosed, metastatic disease; M2, asymptomatic/minimally symptomatic mCRPC that has not been treated with or not progressed on chemotherapy; M3, symptomatic mCRPC that has not been treated with or not progressed on chemotherapy; M4, mCRPC that progressed on/after first-line chemotherapy.

### **Figure A. Incidence of prostate cancer in the United States between 1990 and 2009.** Grouped by clinical state at the time of diagnosis according to the Surveillance Epidemiology and End Results database.

**Figure B. Annual all-cause mortality by clinical state, base-case model in 2009.**

**References**

1. Akaza H, Homma Y, Usami M, Hirao Y, Tsushima T, et al. (2006) Efficacy of primary hormone therapy for localized or locally advanced prostate cancer: results of a 10-year follow-up. BJU Int 98: 573-9.

2. U.S. National Institutes of Health. Surveillance Epidemiology and End Results (2009) National Cancer Institute Web site. Available: <http://seer.cancer.gov/>. Accessed 18 May 2013.

3. American College of Surgeons. National Cancer Data Base (NCDB). Public benchmark reports. Cases diagnosed 2000-2009. (2009) American College of Surgeons Web site. Available: <http://cromwell.facs.org/BMarks/BMPub/Ver10/bm_reports.cfm>. Accessed 18 May 2013.

4. Bill-Axelson A, Holmberg L, Filen F, Ruutu M, Garmo H, et al. (2008) Radical prostatectomy versus watchful waiting in localized prostate cancer: the Scandinavian prostate cancer group-4 randomized trial. J Natl Cancer Inst 100: 1144-54.

5. Moreira DM, Presti JC, Jr., Aronson WJ, Terris MK, Kane CJ, et al. (2009) Natural history of persistently elevated prostate specific antigen after radical prostatectomy: results from the SEARCH database. J Urol 182: 2250-5.

6. D'Amico AV, Hui-Chen M, Renshaw AA, Sussman B, Roehl KA, et al. (2006) Identifying men diagnosed with clinically localized prostate cancer who are at high risk for death from prostate cancer. J Urol 176: S11-5.

7. Shappley WV, III, Kenfield SA, Kasperzyk JL, Qiu W, Stampfer MJ, et al. (2009) Prospective study of determinants and outcomes of deferred treatment or watchful waiting among men with prostate cancer in a nationwide cohort. J Clin Oncol 27: 4980-5.

8. Antonarakis ES, Blackford AL, Garrett-Mayer E, Eisenberger MA (2007) Survival in men with nonmetastatic prostate cancer treated with hormone therapy: a quantitative systematic review. J Clin Oncol 25: 4998-5008.

9. Bolla M, van PH, Collette L, van CP, Vekemans K, et al. (2005) Postoperative radiotherapy after radical prostatectomy: a randomised controlled trial (EORTC trial 22911). Lancet 366: 572-8.

10. Bolla M, Collette L, Blank L, Warde P, Dubois JB, et al. (2002) Long-term results with immediate androgen suppression and external irradiation in patients with locally advanced prostate cancer (an EORTC study): a phase III randomised trial. Lancet 360: 103-6.

11. Antonarakis ES, Chen Y, Elsamanoudi SI, Brassell SA, Da Rocha MV, et al. (2011) Long-term overall survival and metastasis-free survival for men with prostate-specific antigen-recurrent prostate cancer after prostatectomy: analysis of the Center for Prostate Disease Research National Database. BJU Int 108: 378-85.

12. Smith MR, Cook R, Lee KA, Nelson JB (2011) Disease and host characteristics as predictors of time to first bone metastasis and death in men with progressive castration-resistant nonmetastatic prostate cancer. Cancer 117: 2077-85.

13. Tangen CM, Hussain MH, Higano CS, Eisenberger MA, Small EJ, et al. (2012) Improved overall survival trends of men with newly diagnosed M1 prostate cancer: a SWOG phase III trial experience (S8494, S8894 and S9346). J Urol 188: 1164-9.

14. Noguchi M, Noda S, Yoshida M, Ueda S, Shiraishi T, et al. (2004) Chemohormonal therapy as primary treatment for metastatic prostate cancer: a randomized study of estramustine phosphate plus luteinizing hormone-releasing hormone agonist versus flutamide plus luteinizing hormone-releasing hormone agonist. Int J Urol 11: 103-9.

15. Small EJ, Schellhammer PF, Higano CS, Redfern CH, Nemunaitis JJ, et al. (2006) Placebo-controlled phase III trial of immunologic therapy with sipuleucel-T (APC8015) in patients with metastatic, asymptomatic hormone refractory prostate cancer. J Clin Oncol 24: 3089-94.

16. Petrylak DP, Tangen CM, Hussain MH, Lara PN, Jr., Jones JA, et al. (2004) Docetaxel and estramustine compared with mitoxantrone and prednisone for advanced refractory prostate cancer. N Engl J Med 351: 1513-20.

17. de Bono JS, Logothetis CJ, Molina A, Fizazi K, North S, et al. (2011) Abiraterone and increased survival in metastatic prostate cancer. N Engl J Med 364: 1995-2005.
